# Modulation of inflammation by toll-like receptor 4/nuclear factor-kappa B in diarrhea-predominant irritable bowel syndrome

**DOI:** 10.18632/oncotarget.23045

**Published:** 2017-12-08

**Authors:** Xing He, Li-Hong Cui, Xiao-Hui Wang, Zhi-Hui Yan, Chao Li, San-Dong Gong, Yan Zheng, Zhe Luo, Ying Wang

**Affiliations:** ^1^ Third Military Medical University, Chongqing 400038, China; ^2^ Department of Gastroenterology, Navy General Hospital of PLA, Beijing 100048, China

**Keywords:** toll-like receptor 4, nuclear factor-kappa B, diarrhea-predominant irritable bowel syndrome, inflammatory cytokines, pyrrolidine dithiocarbamate

## Abstract

In order to investigate the function of toll-like receptor 4/nuclear factor-kappa B (TLR4/NF-κB) signal pathways in the pathogenesis of diarrhea-predominant irritable bowel syndrome (IBS-D), IBS-D animal models were established in wistar rats challenged with acute and chronic stresses (29 days). Wistar rats without stress-challenged were used as controls. IBS-D models were randomly divided into two groups: one was treated with normal saline, another group was treated with TLR4/NF-κB inhibitor, pyrrolidine dithiocarbamate (PDTC) (50mg/kg/week) for continuous four times. Our results demonstrate that continuous stresses can induce the characteristic symptoms of IBS-D, including high wet stool rate and intestinal flora imbalance. Further examinations of colon tissues show that the protein expression levels of TLR4 and NF-κB in IBS-D groups are higher than that in control group. The secretory levels of interleukin (IL-8), tumor necrosis factor α (TNFα), and myeloid differentiation factor 88 (MyD88) are significantly increased in IBS-D group. Administration with PDTC effectively downregulates levels of these inflammatory factors. In contrast, interleukin-10 (IL-10) is in an opposite alteration with lower levels in IBS-D groups and the PDTC treatment increases it to the levels as in control group. Moreover, inhibition of the TLR4/NF-κB by PDTC improves the microstructure of intestinal mucosa mainly by increasing the height of villi. Our results suggest that TLR4/NF-κB signal pathway plays an important role in the modulation of inflammatory responses in IBS-D, which might be a therapeutic target for the IBS-D. All of these findings also provide the evidence concerning an inherent linkage between the axis of stress/NF-κB/inflammation and IBS-D.

## INTRODUCTION

Irritable bowel syndrome (IBS), a heterogeneous gastrointestinal disorder, is a kind of digestive diseases characterized by chronic recurrent abdominal pain or discomfort associated with abnormal bowel habits and (or) changes of defecation traits. About 15% of people in the world are believed to be affected by this high morbidity rate of disease [1, 2]. According to the changes in bowel habits, IBS is classified into three main types: diarrhea-predominant (IBS-D), constipation-predominant (IBS-C), and alternating stool pattern (IBS-A) [3]. And IBS-D is the most common type [4]. However, it remains unclear the mechanisms to cause IBS-D.

A variety of factors including diet, psychology, gene, environment, stress, and inflammation have been reported to be related with IBS [5–8]. These factors may alter gut-brain axis, induce abnormal gastrointestinal motility, or impair the intestinal mucosal barrier function, subsequently leading to relevant IBS-D symptoms [5–8]. Recently, compelling evidence suggests a possible role for inflammation in the pathogenesis of IBS [9–11]. Inflammatory cytokines, such as IL-1β, IL-8, and TNF-α can affect gastrointestinal motility, secretion, and reabsorption. Additionally, IL-1β and TNF-α can increase IBS-D patients’ intestinal sensitivity [12]. Therefore, finding the mechanisms underlying abnormal expression of these inflammatory factors will definitely benefit IBS-D patients. Notably, the primary mucosal receptors of bacterial components, toll-like receptor family members (TLR4, TLR5, and TLR9) are up-regulated in small bowel mucosa in IBS patients [13]. Furthermore, TLR4 can activate a critical inflammatory mediator, NF-κB to modulate inflammatory responses [14, 15].

In the present study, we sought to specify the role of TLR4/NF-κB signal pathway in IBS-D. Our results demonstrate that a series of stresses can induce characteristic symptoms of IBS-D *in vivo*. Although there is no severe injury in the structure of intestinal mucosa, expression levels of TLR4/NF-κB signal pathway are increased in IBS-D animal model. And the release of inflammatory factors IL-8, IL-10, TNFα, and MyD88 in the serum is also deregulated. Further inhibition of TLR4/NF-κB signal pathway with PDTC can reverse the abnormal inflammatory responses and improve the intestinal mucosal microstructure. All of these results suggest that TLR4/NF-κB signal pathway might be a therapeutic target for IBS-D in clinic.

## RESULTS

### The PDTC treatment relieves the symptoms of IBS-D

**Figure 1:**
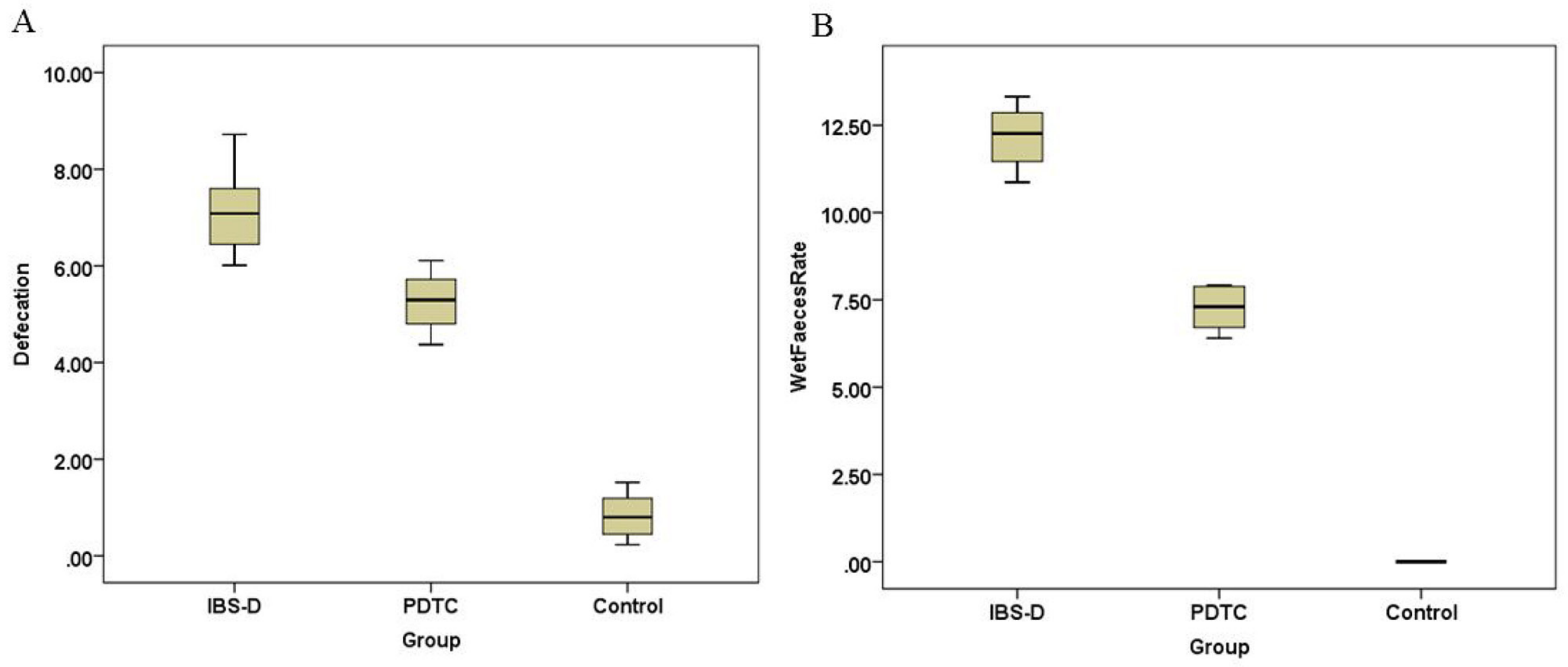
Defecation and wet stool rate in IBS-D, PDTC and control group **(A)** The daily defecation number in IBS-D group was increased when compared with the control (p < 0.05). **(B)** On 30th day, the wet stool rate in IBS-D group was higher than that in the PDTC treated group (p < 0.05).

Diarrhea is a characteristic symptom of irritable bowel syndrome. After challenged with series of stresses, diarrhea appeared earlier in rats of IBS-D group, but not in the control group. The symptom in the PDTC treated group was better than that in non-treated IBS-D group. The daily defecation number in IBS-D group was increased, compared with the control with statistical difference (p<0.05) (Figure 1A). The PDTC treatment could partially control the diarrhea. On 30^th^ day, the wet stool rate in IBS-D group was significantly higher than that in the PDTC treated group (12.15±0.85 versus 7.25±0.58) (p<0.05) (Figure 1B). This result suggests that blocking TLR4/NF-κB pathway may improve IBS-D-related symptoms.

### The expression levels of TLR4/NF-κB are increased in the IBS-D group

**Figure 2:**
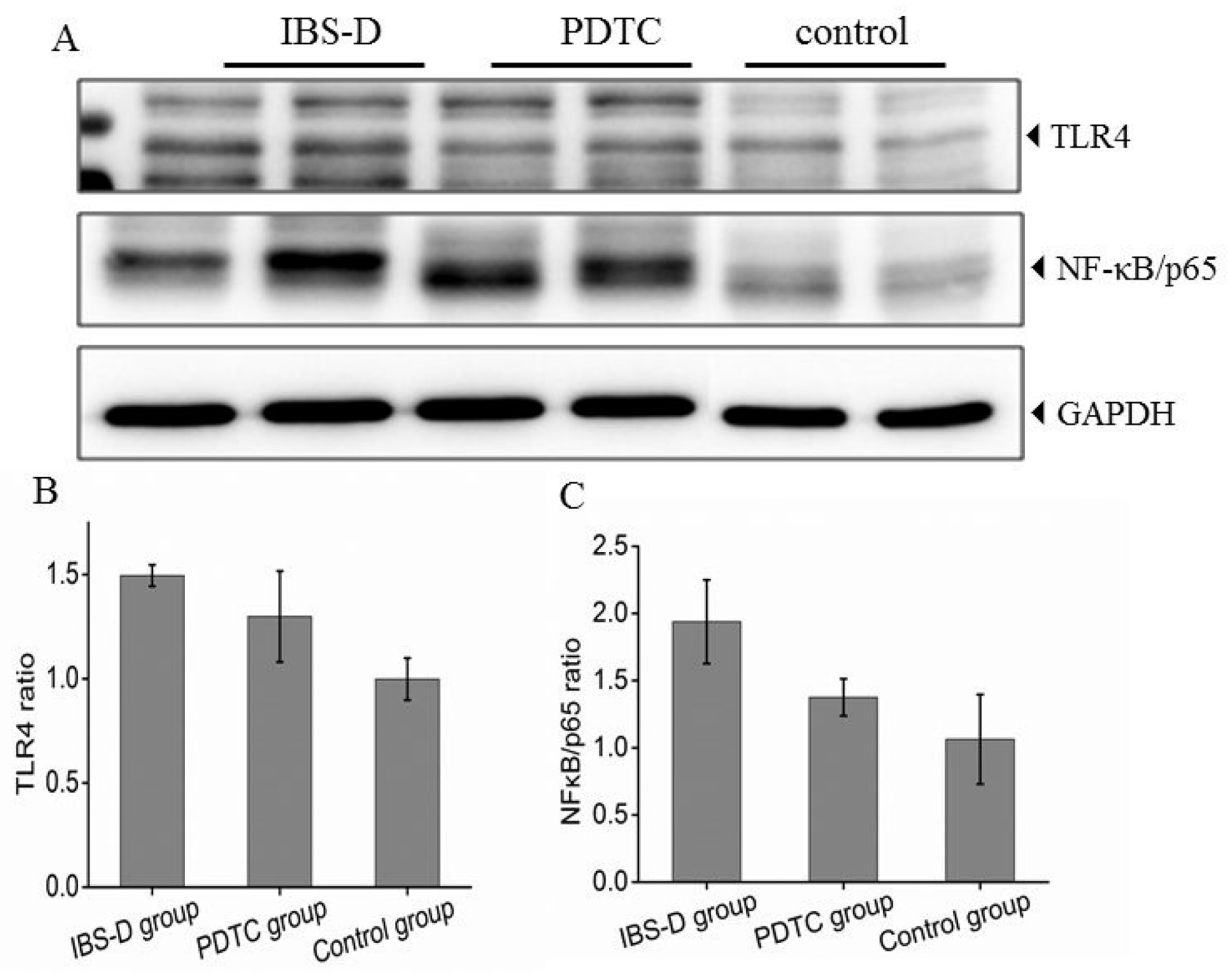
Expression of TLR4 and NF-κB (p65) protein in three groups **(A)** Protein expression of TLR4 and NF-κB (p65) in colon tissues from three groups. **(B)** Histogram of relative gray values of TLR4, *P*<0.05, IBS-D versus control. **(C)** Histogram of relative gray values of NF-κB (p65) in three groups. *P*<0.05, IBS-D versus PDTC.

As shown above in Figure 1, the inhibitor of TLR4/NF-κB pathway has therapeutic effects on IBS-D rats. Further examination demonstrated that protein expression levels of TLR4 and NF-κB in colon tissue were both significantly increased after challenged with stresses (Figure 2A and 2B). The PDTC treatment moderately reduced the expression levels of TLR4 and NF-κB p65 (Figure 2A and 2C), indicating that PTDC modulates TLR4 /NF-κB pathway. The density quantitative results were consistent with the protein expression levels of TLR4 and NF-κB p65 detected by Western blotting (Figure 2B and 2C). The difference between IBS-D and control groups was significant (p<0.05). Elevation of TLR4/NF-κB signal pathway clearly suggests that TLR4/NF-κB may participate in the pathogenesis of IBS-D.

### The PDTC treatment regulates the secretory levels of IL-8, IL-10, TNF-α, and MyD88

**Figure 3:**
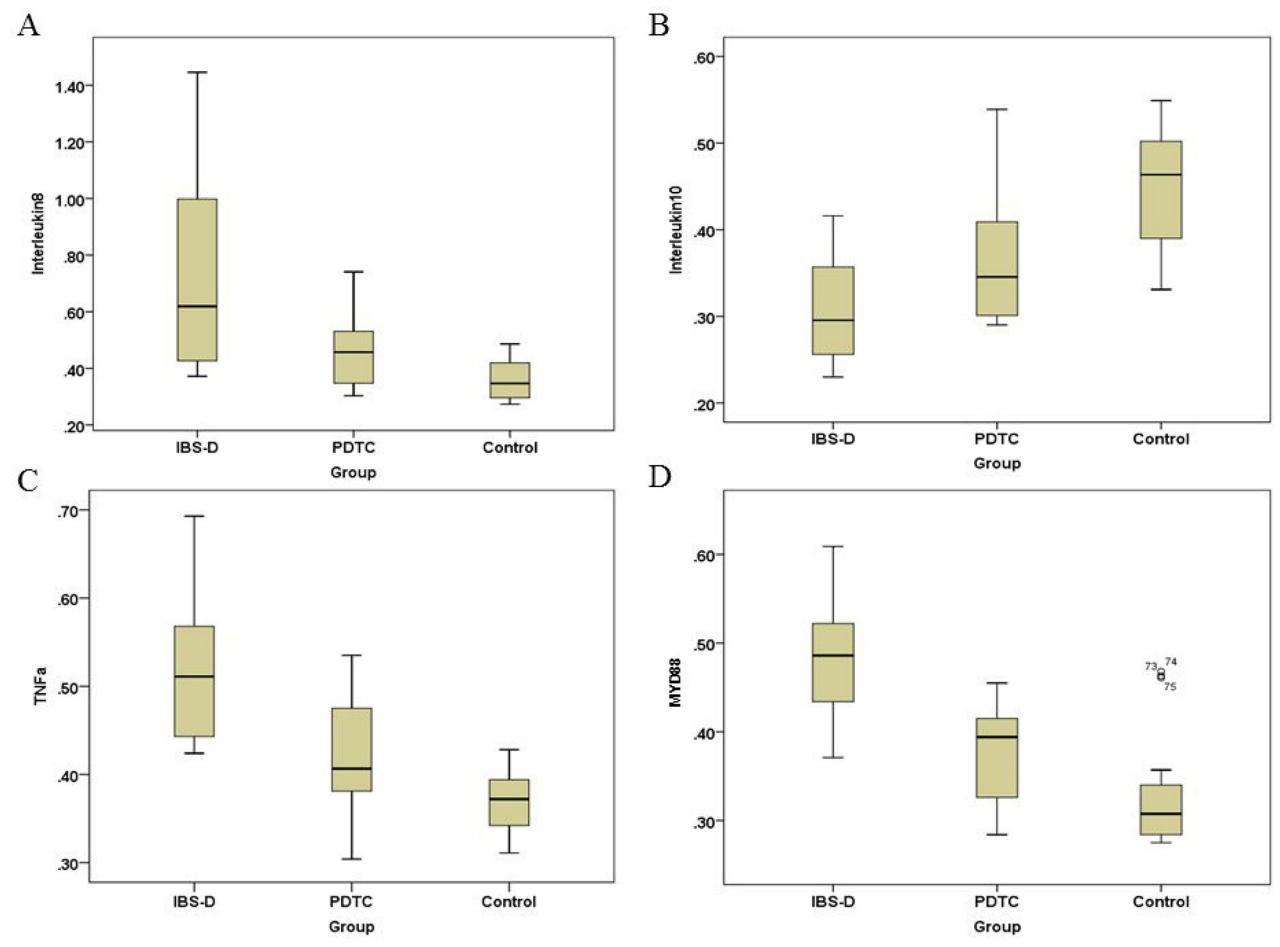
Secretory levels of inflammatory factors IL-8, IL-10, TNF-α, and MyD88 in three groups The levels of inflammatory factors were measured by ELISA. **(A)** Secretory levels of IL-8 in three groups. *P*<0.05, IBS-D compared with PDTC and control, respectively. **(B)** Secretory levels of IL-10 in three groups. *P*<0.05, IBS-D versus control. **(C)** Secretory levels of TNF-α in three groups. *P*<0.05, IBS-D compared with PDTC and control, respectively. **(D)** Secretory levels of MyD88 in three groups. *P*<0.05, IBS-D compared with PDTC and control, respectively.

It is well known that TLR4/NF-κB pathway plays an important role in the regulation of inflammatory responses [14]. To investigate whether inflammation is involved in the pathogenesis of IBS-D, serum samples were collected from three groups of rats. Cytokines were measured by ELISA. Our results showed that secretory levels of IL-8 was significantly increased in IBS-D group when it was compared with that in the control group (p<0.05) (Figure 3A). The PTDC treatment remarkably decreased the levels of IL-8 (Figure 3A). In contrast, the secretory levels of IL-10 was lower in IBS-D than that in the control group (p<0.05) (Figure 3B). There was a trend that the PTDC treatment could upregulate the levels of IL-10, but without statistical difference (Figure 3B). As for the levels of NF-κB-dependent cytokine TNF-α, it was significantly increased in the IBS-D group (p<0.05) (Figure 3C). The PDTC effectively blocked the secretory levels of TNF-α to similar levels as in the control (Figure 3C). The function of myeloid differentiation factor 88 (MyD88) is closely associated with TLR-4 signaling pathway [16]. MyD88 was remarkably increased in the IBS-D group, compared with control (p<0.05). And the PDTC administration was able to reduce the levels of MyD88 (Figure 3D). These results suggest that TLR4/NF-κB pathway can modulate inflammatory factors to affect the progress of IBS-D.

### The PDTC treatment improves the microstructure of the intestinal mucosa

**Figure 4:**
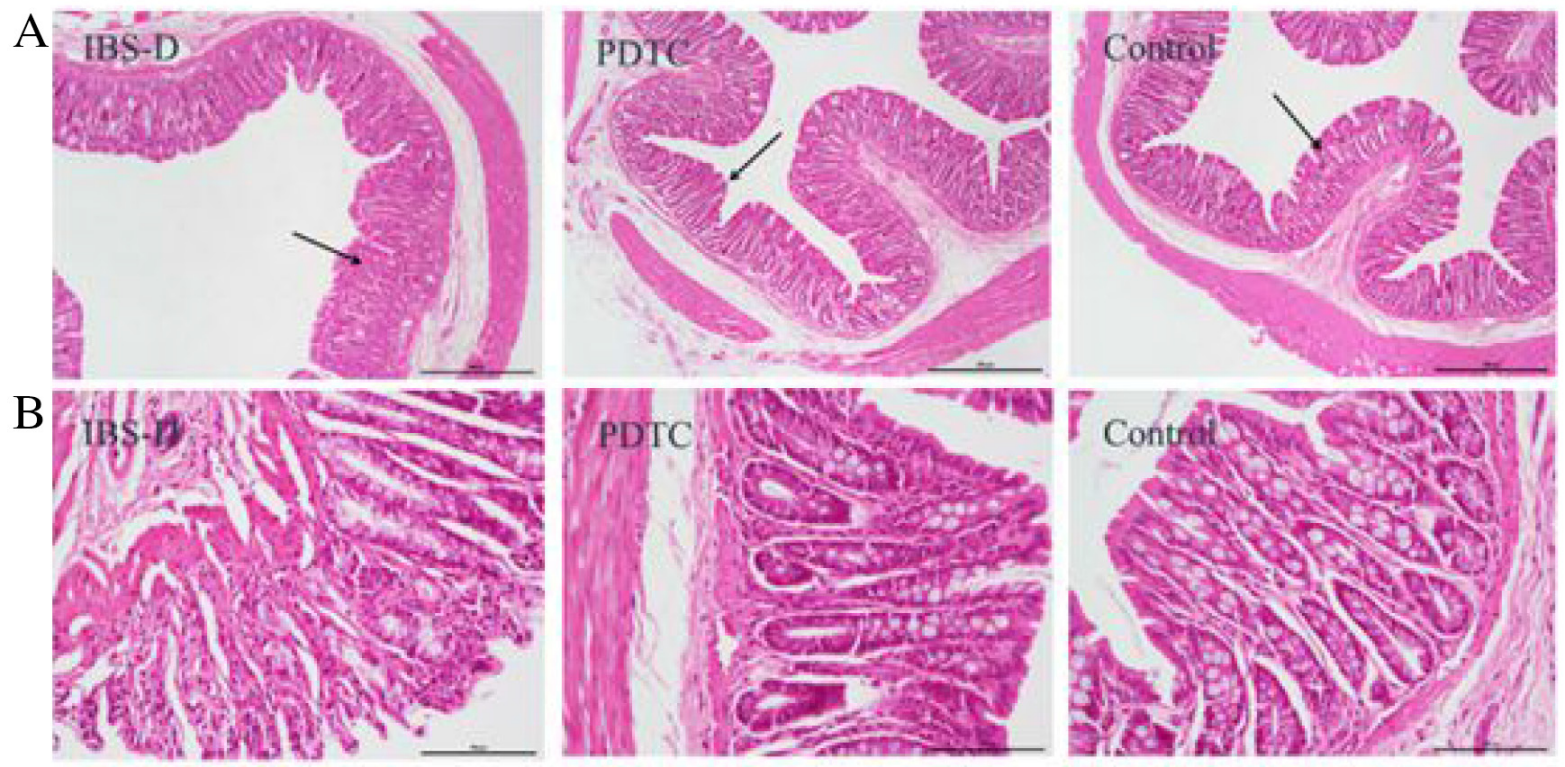
The structure of the intestinal mucosa after H&E staining in three groups **(A)** Images of the intestinal mucosa were taken under light microscope (scale bar equals to amplification fold ×40). **(B)** Images of the intestinal mucosa were taken under light microscope (scale bar equals to amplification fold ×200).

To confirm whether the structure of intestinal mucosa is impaired in IBS-D rats, histological structures of colon tissues from three groups were observed under light microscopy after H&E staining. The intestinal villi were intact and well arranged in control group (Figure 4A and 4B). Generally, there was no severe damage on the intestinal mucosa in IBS-D group. However, some villi loss was observed in IBS-D group (Figure 4A and 4B). The PDTC treatment improved the microstructure changes of the intestinal mucosa with the similar phenotype as that in control group. A clear difference was that the villi height in PDTC group was longer than that in IBS-D group (Figure 4A and 4B). These results suggest there are some damage in colon villi of IBS-D rats which can be relieved by blocking TLR4 / NF-κB pathway.

### PDTC treatment improves the balance of intestinal flora in IBS-D model

**Figure 5:**
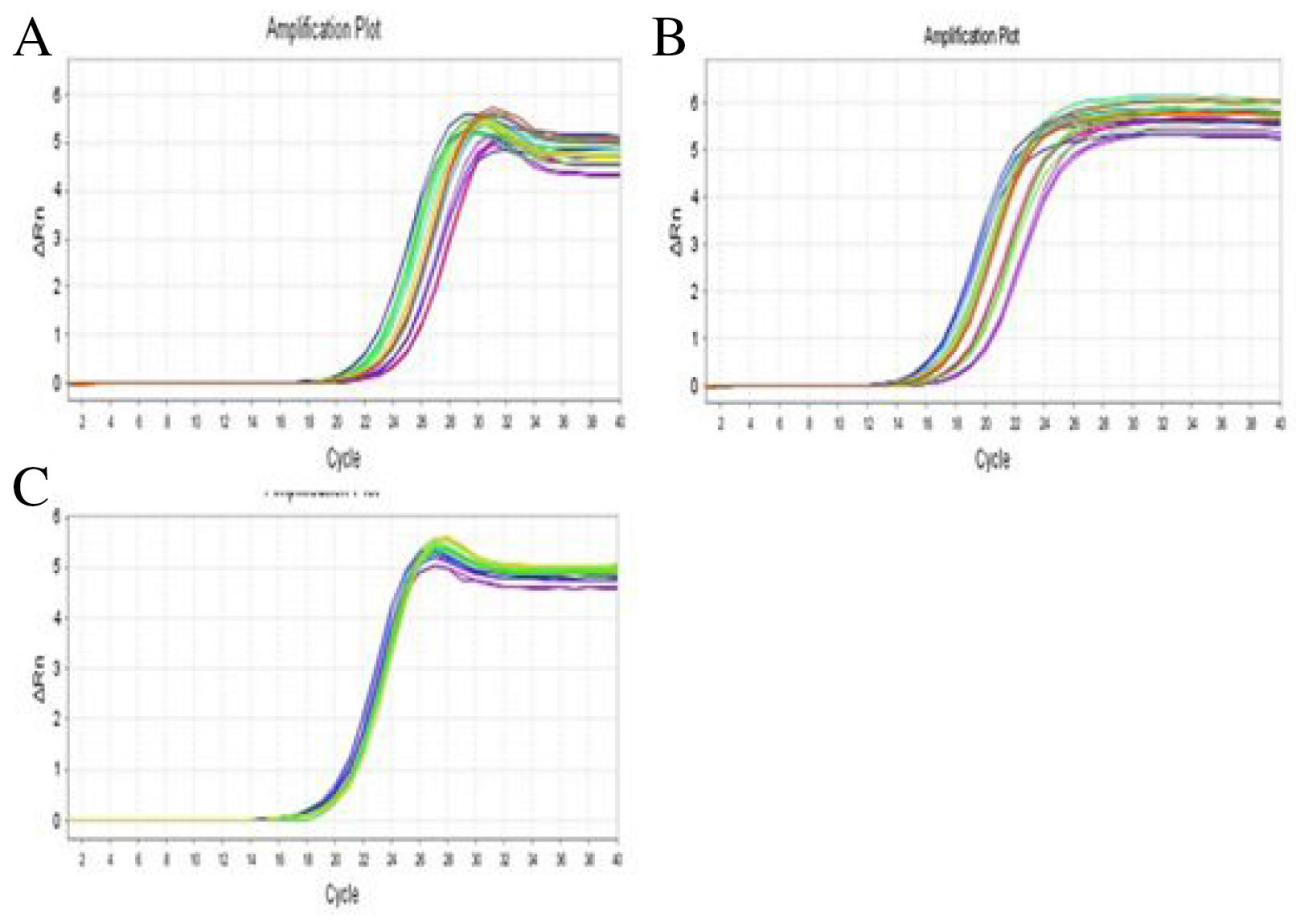
Gene amplification curves of three categories of intestinal bacteria **(A)** Gene amplification curve of *Bifidobacterium*. **(B)** Gene amplification curve of *Lactobacillus*. **(C)** Gene amplification curve of *Escherichia coli*.

**Figure 6:**
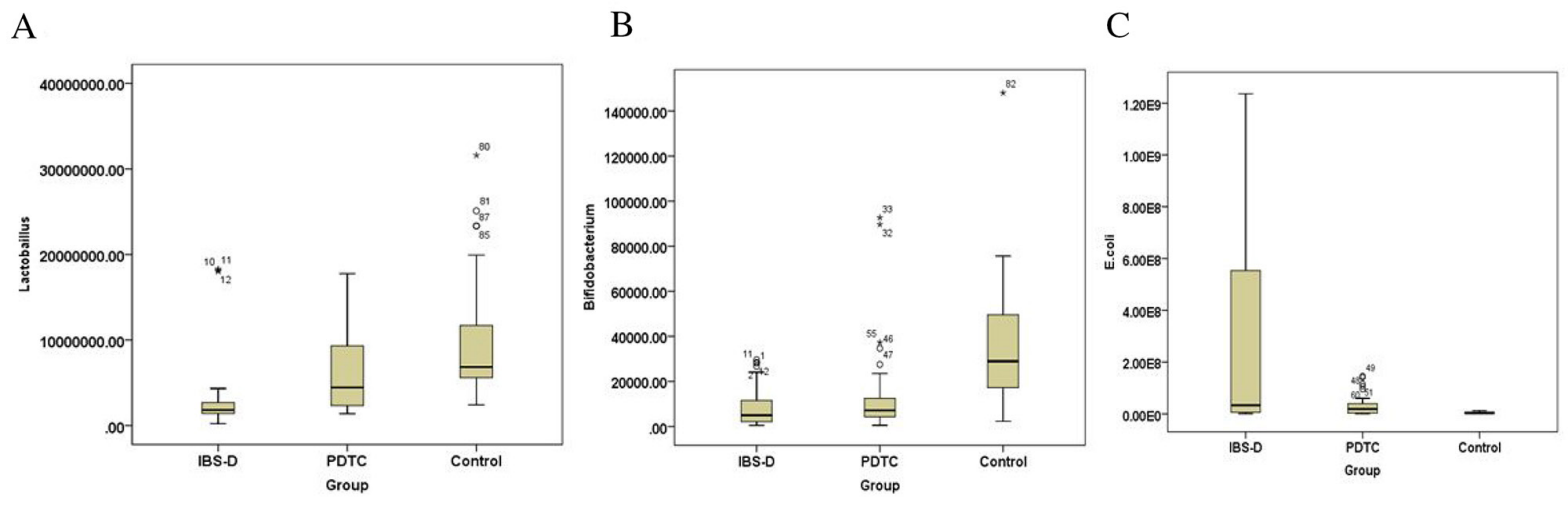
The number of *Bifidobacterium, lactobacillus* and *Escherichia coli* in rats from three groups **(A)** The number of *Bifidobacterium* from three groups. *P*<0.05, IBS-D versus control. **(B)** The number of *lactobacillus* in three groups. *P*<0.05, IBS-D compared with PDTC and control, respectively. **(C)** The number of *Escherichia coli* in three groups. *P*<0.05, IBS-D versus control.

Recent studies have suggested a role for an altered intestinal microbiota in the pathophysiology of IBS [17, 18]. Frequent diarrhea will result in the imbalance of intestinal flora. There are three common categories of microbiota, *Bifidobacterium, Lactobacillus*, and *Escherichia coli* in the intestine. RT-qPCR was performed to detect intestinal bacteria: *Bifidobacterium* (Figure 5A), *lactobacillus* (Figure 5B), and *Escherichia coli* (Figure 5C) in the stool samples from three groups. There was a statistically significant difference among the three groups of bacteria *Bifidobacterium* (F=6.614, P=0.005), *Lactobacillus* (F=7.760, P=0.002) and *Escherichia coli* (F=4.110, P=0.028). Further quantitative results demonstrated that the number of *Bifidobacterium* and *Lactobacillus* in the stool of the rats in IBS-D group were much lower than that in control group (p<0.05) (Figure 6A and 6B), whereas the number of Escherichia coli was much higher than that in the control group (p<0.05) (Figure 6C). After the treatment with the PDTC, the number of *Lactobacillus* was significantly increased, compared with those in IBS-D group (p<0.05) (Figure 6B). There was no significant difference in the numbers of *Bifidobacterium* and *Escherichia coli* between the PDTC group and the IBS-D group (p>0.05) (Figure 6A and 6C). These findings demonstrate that imbalance of intestinal bacteria occurs in IBS-D group and blocking TLR4/NF-κB pathway can partially improve the balance of intestinal microbiota.

## DISCUSSION

The treatment of IBS-D patients is still based on their specific digestive tract symptoms in clinic, due to lacking of a uniform standard[19]. There is an increasing requirement to find an effective target therapy for IBS-D patients. In the current study, we established an *in vivo* model of IBS-D through challenging with stresses. Remarkable elevation of TLR4/NF-κB signal pathway and inflammatory factors were observed in IBS-D rats. Importantly, inhibition of TLR4/NF-κB can reverse the abnormal expression of inflammatory factors and improve the symptoms of ISB-D. Therefore, this study provides an important rationale for the target therapy for ISB-D through blockade of TLR4/NF-κB pathway. All of these findings link the stress/NF-κB/inflammation axis and the IBS-D.

The effects of stress on the pathogenesis of IBS-D have attracted many attentions in patients [20–22]. Researchers are focusing on how the brain-gut axis interaction affects the progress of IBS [23]. Further reduction of stress through psychological interventions has demonstrated therapeutic effects on IBS in clinic [21]. Here, we successfully establish IBS-D animal model after challenging with different stresses, providing a strong evidence to indicate that stresses are causative factors for IBS-D. In response to stresses, many inflammatory cytokines and related signaling pathways are activated, which can affect the function of intestinal mucosa with increasing intestinal permeability or visceral hypersensitivity and eventually result in IBS-D symptoms [24–26], rather than damage the structure of intestinal mucosa. In line with these observations, our results also demonstrate that inflammatory factors, such as IL-8, TNFα, and MyD88 are significantly increased in the serum of IBS-D rats. And these inflammatory factors are modulated by the TLR4/NF-κB signal pathway [27].

NF-κB is well known as a critical transcription factor to modulate both stress and inflammatory responses [28, 29]. Nonetheless, NF-κB can also be activated in the setting of stresses even though the mechanisms are poorly understood [30]. Thus, NF-κB has proven to be a viable therapeutic target for diseases related to stress and inflammation, such as neurodegenerative diseases and diabetes [31, 32]. NF-κB exerts its biological function mainly through dynamic nuclear DNA/protein binding [30]. A variety of inflammatory factors, such as TNF family members are NF-κB-dependent genes. Therefore, inhibition of NF-κB can effectively block the inflammatory pathways. In agreement with these observations, TLR4/NF-κB protein expression levels are elevated in IBS-D model after challenging with stresses. Although we do not observe dramatic downregulation of NF-κB protein levels, NF-κB-dependent TNFα is significantly downregulated by the inhibitor of NF-κB, PDTC, indicating that the activity of NF-κB is effectively blocked by this inhibitor. Notably, TNFα has been considered as a key inflammatory factor relevant to IBS [33]. Importantly, the symptoms such as diarrhea and microstructure of mucosa are improved by the NF-κB inhibitor treatment in our IBS-D model.

Collectively, our findings indicate a correlation between the stress/NF-κB/inflammation axis and IBS-D. NF-κB functions as a key mediator to modulate stress and inflammatory responses. It is worthy to mention that multiple factors are involved in this stress-associated inflammatory cascades [34]. All of these molecules form a complex network to regulate the progress of disease. Our data provides an important rationale for further exploring target therapy in IBS-D.

## MATERIALS AND METHODS

### Materials

Pyrrolidine dithiocarbamate (PDTC) was purchased from Sigma-Aldrich. TLR4 antibody was obtained from Bioss (USA), NF-κB antibody was bought from Abcam (USA), and GAPDH antibody was from ZSGB-BIO (China).

### General animal experiments conditions

Animal experiments were conducted in animal room with Specific Pathogen Free (SPF) standards. All animal experiment protocols were reviewed by Institutional Animal Care and Use Committee. The experimental animals were SPF female Wistar rats at average weight (160 ± 10g) and were purchased from Ke Yu animal breeding center (Beijing, China). The animal experiments were performed in the Center laboratory in Navy General Hospital (Beijing, China). All rats used for experiment were kept for 3 days rest (d), then were housed in separate plastic cages (25×25×35cm) for 1 week (w) of acclimation. During the experiment, rats were provided with the standard diet, tap water, cages’ temperature of 22 ± 2°C, and 12h/12h lighting / dark rhythm. This animal experiment protocol was approved by Ethics Committee of Navy General Hospital (Beijing, China).

### Establishing IBS-D animal model and treatment

Total 30 rats were randomly divided into three groups: IBS-D group, PDTC group, and control group. The rats of IBS-D group and PDTC group received acute and chronic stresses to build up IBS-D model (4 week). Specific stresses [23] included 12 hours of continuous lighting at night, 45°C hot environment for 5 minutes, 4°C cold environment for 3 minutes, tail clamp for 1 minute, horizontal vibration (120 / min) for 40 minutes, and food deprivation for 24 hours. The Wistar rats in IBS-D group and PDTC group experienced the mentioned stresses once a week in the random order for 3 consecutive weeks and then rested 1 week. On the 29th day, their anterior shoulder, the forearm and the chest were bound by tapes for 1 hour to restrict the upper limb scratching the head and face, but not to restrict their activities. The only different procedure between IBS-D group and PDTC group is that the rats in IBS-D group received one time intraperitoneal injection of normal saline (1ml per week) for 4 times, while the rats in PDTC group received once a week of intraperitoneal injection of PDTC (50mg/kg) [15] for four times. Rats in control group did not take any operation. The wet stool rate and sucrose water intake of the mice were recorded on 30th day.

### Wet stool rate

Wet stool rate was calculated daily by filter paper imprint to determine the starting time of diarrhea, the total number of defecation within 24 hours, and the number of wet stool. Wet stool rate=the number of wet stool/the number of defecation×100%.

### Samples collection and processing

The fecal samples were collected on 30^th^ day of experiment and immediately stored at −80°C for RT-qPCR analysis. On 31^th^ day of experiment, all the rats were anesthetized. Blood samples were collected through inferior vena cava. After centrifuge, serum was collected and stored in −80°C for cytokines detection. Then, rats were sacrificed. A 4cm long sample was taken from each Sigmoid colon (about 8cm distal to the anus), 1 cm from two ends were removed respectively. The rest of 2cm gut tissue was cleaned with physiological saline and divided into two parts: one part was fixed in 10 % formalin for Hematoxylin & Eosin (HE) staining, another part was stored in liquid nitrogen for Western blotting.

### Western blotting

Intestinal tissues were collected from three groups of rats. Protein was extracted with lysis buffer. Total amounts of 30μg protein per sample were separated by electrophoresis, then were transferred onto PVDF membranes (Invitrogen, USA). The membranes were probed with antibodies against to TLR4 and NF-κB (p65). GAPDH was used as a loading control.

### Histological analysis of intestinal tissue

Paraffin-embedded intestinal tissues were cut into 5-mm-thick sections. The sections were immersed in xylene and hydrated with ethanol to deparaffinize. These sections were routinely processed for HE histology. The Villus structure, length and integrity of gut mucosa were observed by optical microscope (Olympus, CHZ12). Images were taken by digital camera (JEM-1230, Japan).

### Quantitative real-time reverse transcription-PCR

The fecal flora was determined by quantitative RT-PCR. The genomic DNA in the fecal samples was extracted by the Genome DNA Extraction Kit (DP328) (TIANGEN BIOTECH, China). RT-qPCR reactions were carried out using an ABI (Applied Biosystems) and the SYBR® Premix Ex Taq™ II with ROX plus. Species-specific primers for the quantification of 16S rDNA gene belonging to Bifidobacterium, Lactobacillus and Escherichia coli were used as previously reported [35, 36] and synthesized by Invitrogen. Bifidobacterium primers sequence: forward 5′-GATTCTG GCTCAGGATGAACGC-3, reverse 5′-CTGATAGGACGCG ACCCCAT-3′; Lactobacillus primers sequence: forward 5′-AGCAGTAGGGAATCTTCCA-3, reverse 5′-CACCG CTACACATGGAG-3′; Escherichia coli primers sequence: forward 5′-CGGCAACGAGCGCAACCC-3′, reverse 5′-CCATTGTAGCACGTGTGTAGCC-3. The amplification involved one cycle at 95°C for 30s, followed by 40 cycles of denaturation at 95°C for 15s, 40s at different annealing temperature [14] (Bifidobacterium 55°C, Lactobacillus 50°C, Escherichia coli 52°C), extension at 72°C for 45s. Standard curves for each strain were constructed using DNA extracted from microbial cultures using tenfold dilutions ranging from 1010 CFU/mL to10 CFU/mL.

### Enzyme-linked immunosorbent assay (ELISA)

Levels of inflammatory cytokines (IL-8, IL-10, TNF-α, and MyD88) in serum were measured using ELISA kits (Boster, China) according to the manufacturer’s protocols. The results were recorded as picograms (pg) of IL-8, IL-10, TNF-α, and MyD88 per ml of serum.

### Statistical analysis

All data were analyzed by SPSS20.0 software. Differences among groups were analyzed by one-way ANOVA. Least significant difference (LSD) method was used for multiple comparisons. Two group comparisons were carried out using a two-tailed Student’s *t* test. When *P*<0.05, the difference was considered as statistically significant.
